# Exploring College Students’ Compliance During a 60-Day Alcohol-Related Ecological Momentary Assessment Using Location- and Time-Based Questions: Prospective Observational Study

**DOI:** 10.2196/81713

**Published:** 2026-10-09

**Authors:** Lucy E Napper, Bilal Khan, Gavin Q Fox, Mohamed K Saad, Laura C Wolter, Dennis E McChargue

**Affiliations:** 1 Department of Psychology, Health, Medicine and Society Program College of Arts and Sciences Lehigh University Bethlehem, PA United States; 2 College of Health Lehigh University Bethlehem, PA United States; 3 Department of Psychology University of Nebraska-Lincoln Lincoln, NE United States

**Keywords:** Alcohol Consumption, Compliance, Feasibility, Acceptability, Ecological momentary assessment, College, Mobile sensors, Privacy

## Abstract

**Background:**

Ecological momentary assessment (EMA) methods can provide assessment of alcohol-related beliefs and behaviors when people are in their natural environments. An increasingly common EMA approach involves the use of passive sensors (eg, continuous sharing of Bluetooth and GPS cell phone data) to collect rich data and trigger alcohol assessment in specific contexts. While collecting real-time assessments allows researchers to explore contextual factors impacting alcohol use, there is a lack of research examining compliance rates to alcohol-related EMAs that use daily questions for study periods over 2 weeks. EMAs over longer periods allow researchers to assess meaningful changes over time and sample low-base-rate events. Further, there is limited research exploring how EMA compliance is influenced by individual-level factors (eg, privacy concerns and alcohol use). Identifying factors associated with compliance can help inform EMA protocol design.

**Objective:**

This study had 2 primary goals. First, we add to the literature by examining the feasibility and acceptability of a 60-day college student alcohol-EMA that uses phone-based, passive sensors. Second, we examined whether compliance was associated with event-level alcohol use, privacy concerns, and types of questions asked (ie, questions triggered by time or GPS location).

**Methods:**

College students (N=68) who reported regular alcohol use completed the 60-day EMA study. At baseline, participants completed an online survey that included an assessment of privacy concerns. During the study period, participants completed up to 15 questions a day, including evening and morning self-reports of alcohol use. Feasibility was assessed based on enrollment, retention, and compliance rates. Acceptability was examined at the end of the study period using open- and close-answered questions on perceptions of the EMA experience.

**Results:**

The findings supported the feasibility of the 60-day EMA with enrollment exceeding benchmarks and high compliance across the study period (average compliance=90%). Overall, students found the protocol acceptable with 88% (58/66) indicating that they would be willing to complete another EMA study. Participants’ compliance was not associated with alcohol use at the event-level (odds ratio [OR] 0.94, 95% CI 0.80-1.10) or at the trait level (OR 0.92, 95% CI 0.73-1.17). Participants’ compliance was greater for time-trigger assessment than GPS-triggered questions (*P*<.001). Privacy concerns were not associated with reduced responding.

**Conclusions:**

This study has implications for those designing alcohol EMAs for college populations that involve the use of passive sensors, including recommendations for balancing predictable timing of questions and variability in question content.

## Introduction

### Background

Heavy alcohol use is an issue of concern among college students, with 21.9% of students reporting binge drinking [1]. Alcohol use is associated with a range of negative consequences both for students who drink (eg, unintentional injuries, problems with friends, and interference with schoolwork) and the broader community (eg, damage to property and interrupted sleep) [2,3]. Ecological momentary assessments (EMAs) offer one approach to understand proximal predictors of high-risk drinking behaviors, test theoretical models of alcohol use, and inform the development of just-in-time interventions targeting heavy alcohol use.

EMAs involve real-time assessment of beliefs and behaviors as participants go through their daily lives (ie, in their natural environment) [4]. EMAs are a broad category of approaches that vary in format (eg, text messaging–based vs app-based). In comparison to traditional survey methods assessing retrospective reports of behaviors and beliefs at a single time point, EMAs have a number of advantages. For instance, EMAs increase ecological validity and allow researchers to study dynamic processes that influence drinking, the influence of context on alcohol beliefs and behaviors, and temporal associations between constructs of interest [4,5]. EMAs are well suited to study alcohol use among college students because drinking often happens during discrete events [5]. Indeed, there is a growing body of alcohol-focused research using EMAs to study craving [6], drinking motives and decisions [7-9], negative affect or anxiety [10,11], impact of exposure to alcohol marketing [12], and social context [13,14] in college populations.

In order for EMAs to produce valid and useful data, participant compliance is essential. Work on alcohol-related EMAs among college students has produced compliance rates between 63.8% [6] and 96.8% [8]. There is a growing body of work, including meta-analyses, examining factors that might explain variation in EMA protocol compliance across different research topics, protocols, and samples [15-18]. One factor that might impact compliance is the length of the protocol. In the context of noncollege populations, alcohol-related EMAs over short periods (2 to 4 weeks) have generally been found to be acceptable and feasible, with participants describing EMAs as easy to complete and nondisruptive [19-21]. In contrast, there are mixed findings on the feasibility of alcohol-related EMAs over longer periods. Sokolovsky et al [22] found a steady increase in missed questions across a 56-day EMA divided into 2 bursts. Similarly, a 14-week EMA study found compliance rates significantly dropped over time (93% to 39%) [23]. While another college-based study found less pronounced reductions (90% to 70%) [24]. These inconsistencies in the college alcohol literature suggest that additional research exploring protocols greater than 14 days would be beneficial. Furthermore, Wrzus and Neubauer’s [16] recent review of EMAs did not identify a single study that used more than 10 prompts per day over a period of greater than 14 days, highlighting the need for more studies of this type.

In addition to length of data collection, other aspects of an alcohol-related EMA protocol may impact compliance. For instance, Hultgren et al [25] examined compliance to both daily questions administered at set times and event-contingent momentary assessments initiated by participants on drinking occasions. They demonstrated that compliance was greater for regular daily questions than more irregular, self-initiated EMA questions. Differences in compliance might reflect the ease of remembering to respond to questions at set times or challenges to completing questions once alcohol use is initiated. Indeed, event-contingent momentary assessments were less likely to be completed when students were drinking more than usual for them. Similarly, Suffoletto et al [23] note that intoxication negatively impacts completion of reaction time–based EMA tasks. However, the relationship between alcohol use and EMA compliance remains unclear. For instance, other research suggests that neither same-day or past year alcohol use are associated with EMA compliance [24,26].

Other individual-level predictors may be important for understanding protocol compliance in EMA studies, but this type of research to date has been limited [26]. In a college-based alcohol-related EMA, Howard and Lamb [24] explored demographic, personality, and well-being variables and found no associations between these variables and compliance. In non-EMA survey studies, one factor that may be related to missing data is participants’ privacy concerns [27]. These concerns might be particularly relevant to understanding compliance in the context of EMA studies examining sensitive topics, such as substance use, or for EMA approaches perceived as more invasive. For example, GPS and Bluetooth data can be integrated into EMAs in order to address novel questions about the environmental and social context of drinking and help identify risk factors for alcohol use beyond individual-level factors (eg, mood). In addition, integrating sensor data allows questions to be triggered based on, for example, someone’s geographic location. While incorporating mobile sensors into EMAs is not common, representing about 5% of EMA studies [15], this approach is likely to become increasingly popular given the opportunities that it provides for studying contextual factors. That said, it is possible that both GPS tracking and triggering questions based on a participant’s geographic location may raise privacy concerns [28,29]. Therefore, research is needed to determine if privacy concerns impact compliance in these types of EMA studies.

While research has examined the acceptability of traditional EMA techniques, there is less known about participants’ perceptions of EMAs that include passive sensing methods that occur automatically and continuously [30] and how individual-level factors impact compliance for this approach. Further, based on a recent review of EMA including geographic technology [31], no studies to date have specifically explored the use of EMAs that incorporate GPS-based sensors when examining college student alcohol use. Therefore, this study extends past research by examining the feasibility and acceptability of a 60-day, alcohol-related EMA that required participants to share Bluetooth, motion and fitness sensor, and GPS cell phone data.

### Current Study

This study has 2 primary goals. First, we aim to assess the feasibility (eg, based on enrollment, retention, and overall compliance to daily questions) and acceptability (eg, participants satisfaction with the protocol) of a 60-day EMA alcohol-related protocol. While EMAs have the potential to advance our understanding of the proximal predictors of alcohol-related outcomes, they will only do so if participants are willing to enroll in and complete EMA protocols. This study adds to the limited research using daily surveys over periods greater than 14 days. Testing the practicality of these types of longer EMA designs is important for a number of reasons. For example, longer EMAs are important both for understanding alcohol-related risk factors that unfold and meaningfully change over weeks rather than days (eg, shifts in mood trajectories, coping patterns, and intervention response). As such, longer assessment windows are critical to observe within-person change instead of short-term variability. Moreover, longer assessment periods increase the chance of sampling low-base-rate events (eg, rare stressors and high-intensity drinking episodes) that are essential for testing risk models and just-in-time intervention assumptions. In the context of college drinking, this study’s focus on a 60-day time frame allowed us to test feasibility and collect rich data across different phases of the semester including multiple weekends, midterms, and Spring break.

The second aim of the paper is to explore whether individual-level and EMA-design elements impacted compliance. Identifying factors that could impact compliance is important for understanding potential data biases and for improving EMA protocol design. This study adds to the literature by examining whether compliance rates were related to (1) students’ alcohol use, (2) students’ privacy concerns, and (3) EMA question type (ie, daily time-based question vs geolocation-triggered question). Based on past research [25], we expected compliance to be greater for time-based vs geolocation-based questions. Given the lack of data on compliance and privacy concerns, and the mixed findings on the relationship between alcohol use and compliance, these analyses were exploratory.

## Methods

### Procedures

A random sample of students from a university in the Northeastern United States (N=399) were invited to participate via email that provided a link to an online screening survey. In addition, participants were given the option to invite friends to participate in the study either by providing the researcher with their friend’s email address or by the student sharing a link to the study. This approach was used to increase the likelihood of phones being collocated in order to maximize the amount of Bluetooth data collected. Eligibility was determined based on cell phone ownership, being 18 years of age or older, not planning to study abroad during the study period (n=1 excluded), and drinking at least twice a week (n=137 excluded). Of the 107 eligible participants, 84 expressed interest in participating, completed an online baseline survey, and provided contact information. Participants were enrolled in the study until our recruitment goal (n=75) was reached and remaining students were placed on a waitlist (n=9). Participants were scheduled to attend a brief meeting (in-person or on Zoom [Zoom Communications, Inc]) to receive more information about the study and the EMA app (called ODIN; Grouptheory Inc). Participants were guided through installing the ODIN app on their personal phones and received information about use of passive sensors and how to adjust their phone settings to allow or suspend access. Participants were then shown what to expect while using the app, including example questions, how to track their own participation, and how to contact the research team through the app.

The final study sample (N=68, 91% of those initially enrolled; 68/75) includes participants who attended a training meeting and installed the ODIN app (41/68, 60% recruited directly; 25/68, 37% through friend referral). Participants completed daily questions via the ODIN platform over a continuous, 60-day period between February and April. At the end of the 60 days, participants were sent an email to complete a follow-up survey.

### Ethical Considerations

All procedures were approved by the Lehigh University Institutional review board (protocol #1940662-5). All participants provided informed consent. EMA and survey data were anonymized and linked via a random number. Participants received US $20 for completing both the baseline and follow-up survey. In addition, participants could earn up to US $3 a day based on the percentage of questions they answered (eg, a participant who received 10 question prompts and responded to 5 would receive US $1.50).

### Participants

The majority of the participants were women (45/68, 66%; 22/68, 32% men; 1/68, 1.5% nonbinary) and were aged between 18 and 23 (mean 20.4, SD 1.2) years. In terms of race, 78% (53/68) identified as White, with 9% (6/68) multiracial, 6% (4/68) Black or African American, 6% (4/68) Asian, and 2% (1/68) Native Hawaiian or Pacific Islander. Further, 9% (6/68) of the sample identified as Latine. At baseline, on average, students reported drinking 17 (SD 11.7) drinks per week. Most participants had smartphones that ran on iOS (65/68, 96% Apple Inc; 3/68, 4% Android users, Google LLC ) and at baseline reported using their phone approximately 263.5 minutes a day. Prior to the EMA period, 67 of the 68 (99%) participants reported using their cell phone when out at a party or bar, with the most frequent usages being texting and/or instant messaging and taking pictures and/or video.

### Event-Level Procedures

#### Overview

Questions varied by day of the week, with participants receiving up to 15 questions per day. This included a combination of questions triggered by time (eg, drinking plans reported at noon Thursday to Saturday) or GPS location (see below for more details). Participants received between 2 and 4 time-based surveys a day (plus additional surveys triggered by GPS location). Each survey consisted between 1 and 6 questions. In order for participants to receive questions, the ODIN app had to be running on their phone. If participants closed the app, they would receive a notification asking them to reopen it. If a participants’ app was closed for ~3 days, they received up to 4 emails inviting them to continue their participation.

#### Passive Sensors

The ODIN app uses Bluetooth Low Energy (BLE) protocols on iOS and Android to ensure that each participant’s app continuously broadcasts their assigned participant identification number, albeit encrypted by the study-wide secret key. This allowed us to detect when participants were close to other phones that were running the ODIN app. We continuously collected geolocation data (~every 5 m). In order to optimize battery life, motion sensor data was monitored, but not recorded. More specifically, when motion sensor information determined that a participant had not moved from the previous recorded GPS location, GPS data was not recorded.

#### Morning Retrospective Report

Every morning (9 AM) participants were asked about the previous night including if they attended a social gathering where drinking occurred, how many alcoholic drinks they and others consumed, and what harm reduction strategies they used. The morning self-reported drinking item was used to predict compliance to questions presented the prior evening. Participants also indicate if and when they became too intoxicated to respond to ODIN questions (response option: “No [were never too intoxicated],” “Yes, by 8PM,” “Yes, by 10PM,” “Yes, by midnight,” and “Yes, after midnight”).

#### Time Triggered Questions

In order to assess compliance during common drinking times, participants received a question at 10 PM (Thursday-Saturday) that varied in content (eg, a word unscramble task or being asked to locate someone close by who had a specific characteristic). At 10:30 PM (Thursday-Saturday) participants also received alcohol-related questions, “Are you currently with other students?” “Are you or people around you drinking alcohol?” “What is the MAXIMUM number of drinks it would be acceptable for a typical student to drink at the place you are at?” and “How many standard alcoholic drinks have you had today?” In addition to alcohol questions, participants received nonalcohol related questions related to sense of belonging at college (Sunday-Wednesday at 4 PM). For example, participants were asked to indicate the extent to which “I feel at home in this space.”

#### Geolocation-Triggered Questions

On Thursdays through Saturdays, ODIN delivered geolocation-triggered questions when a participant’s phone remained within a prespecified circular geofence surrounding a target drinking venue for at least 10 minutes. The research team identified seven bars and clubs that served alcohol and were located within approximately 0.5 miles (0.8 km) of campus. Target venues were identified using local knowledge and verified using Google Maps (Alphabet Inc), Google Maps venue listings, and the latitude and longitude associated with each venue’s Google Maps place pin were used as the center of the geofence. The 0.5-mile distance was used as a practical criterion for selecting target venues; it was not the spatial buffer used to trigger questions. Prompt delivery was instead based on a fixed circular buffer with a radius of 100 feet (30.5 m) around each venue coordinate. For each venue, prespecified triggering hours were determined based on when each establishment was open. Before study launch, research staff visited each target location and confirmed that a prompt was generated after the 10-minute dwell criterion was met. Prior research using ODIN documented high completeness of GPS acquisition over a 30-day period [32]. The geolocation-triggered survey asked participants whether they had been or were currently at a location where alcohol was served.

### Survey Measures

#### Baseline Survey

Prior to EMA data collection, participants completed 3 baseline measures of privacy concerns: location privacy, general privacy risk awareness (adapted from [33]), and concern for information privacy (CFIP; [34]). For all privacy measures, participants indicated their level of agreement on a 7-point scale (1=strongly disagree, 7=strongly agree). Location privacy consisted of 4 questions that began with the stem, “I am fine with sharing my location with…,” followed by different types of apps (ie, map, weather, camera, and shopping apps) as well as one question regarding location sharing in general (ie, “In general, I am comfortable sharing my location information on my smartphone, Cronbach α=–0.87). General privacy risk awareness consisted of three questions (ie, “I am aware of potential privacy threats and their consequences from using my smartphone,” “I am aware of possible privacy breaches from using my smartphone,” and “I understand the privacy risks from using a smartphone,” Cronbach α=0.91). The CFIP consisted of 15 questions (Cronbach α=–0.91) assessing participants’ concerns that corporations were misusing their data and other private information, with higher scores indicating more concern.

#### Follow-Up Survey

At the end of the 60-day EMA, participants completed measures of acceptability (based on [35]), including general perceptions of study protocol [36,37] and emotional responses to using the EMA (eg, interest, enjoyment, and annoyance [37]). For example, participants were asked questions related to ease of use: “How did using the ODIN app fit into your routine?” and “How easy was it to answer the daily questions?” (1=very easily to 5=very difficult). Overall satisfaction was assessed with items such as, “Overall, how satisfied are you with the app you used over the last 60 days?” (1=very satisfied to 5=very dissatisfied) and “Would you be willing to take part in another ODIN study that involves daily surveys?” (1=no, definitely not, to 4=yes, definitely). After individual items, open-ended prompts (“please explain,” “why or why not?”) were used to clarify responses. Participants were also asked to describe any problems (What problems or challenges did you encounter using the ODIN app throughout the study?) or benefits experienced while using the app (Did you experience any benefits from using the ODIN app?). Finally, participants were provided with a list of potential difficulties in maintaining answering the daily questions and asked to indicate which applied to their experiences. The list included privacy concerns (ie, type or quantity of data collected and worries about data safety), social reasons (ie, busy schedule, interfering with schoolwork), question-related issues (ie, too many questions, too often, or at inconvenient times), and technical issues technical issues (ie, app did not work, app crashed).

At follow-up, participants were also asked to describe their alcohol use over the 60-day EMA period both in terms of the average number of drinks they had on each occasion and how many drinks they had on their maximum drinking occasion [38].

### Analysis Plan

#### Feasibility and Acceptability

Feasibility and acceptability were evaluated using proportions. Benchmarks for feasibility included ≥50% enrollment, ≥70% of participants retained (ie, still engaged with the app at the end of the 60 days, and ≥70% response rate to daily questions [39-41]. Our target benchmark for acceptability was ≥80% endorsement of statements supporting satisfaction with participants’ experience with study protocol. In addition, we examined whether participants’ emotional responses to using the app (interest, fun, and annoyance) significantly differed from the neutral midpoint of the response scale (4). Open-ended responses were reviewed by 2 authors and summative content analysis was used to identify common categories of responses.

#### Associations With Compliance

Overall compliance rates were calculated separately for each person by adding the number of surveys completed, dividing this number by the number of surveys received, and multiplying by 100. We examined whether the overall measure of compliance was correlated with privacy concerns assessed baseline. To address whether compliance differed based on sensor type used to trigger questions, we conducted a paired 2-tailed *t* test comparing mean compliance rates for 2 alcohol questions that were triggered by GPS location (bars during opening hours) and time of day (10:30 PM) on Thursday, Friday, and Saturday nights.

To examine the association between compliance rates and alcohol use we examined model fit for generalized linear mixed-effects models using a range of distributions and link functions. Given rates of compliance were generally high and there was little variability (318/362, 88% of the time participants were 100% compliant), based on residual diagnostics and model fit information, we determined that dichotomizing the data (0=not compliant [less than 100%], 1=100% compliant) was the most appropriate analysis strategy. We examined the relationship between morning reports of total alcohol consumed the night before with compliance rates from the night before (for all time-based and GPS trigger questions answered between 10 PM and 2 AM) fitted with a logistic mixed model estimated using maximum likelihood using the glmer function from the *lme4* package in R (version 4.3.1; R Core Team). Fixed effect coefficients were exponentiated to obtain odds ratios (ORs) and associated 95% CIs. Responses on occasions where participants did not drink were not included in these analyses. We examined both whether state- (alcohol use on a given night [person-centered]) and trait-level drinking (average alcohol use on occasions when they drank [grand mean-centered]) predicted compliance while accounting for random variability between participants.

#### Data Quality

As per Checklist for Reporting Ecological Momentary Assessment Studies (CREMAS) guidelines [42] for reporting EMAs, we describe information about data quality. This includes information on participants’ response latencies to question prompts and the minimum number of prompts planned and delivered. In addition, we explore preliminary support for the concurrent validity of participants’ responses by examining the correlations among alcohol use reported in the EMA to retrospective reports based on the post-EMA online survey.

## Results

### Feasibility

#### Overview

We exceeded our benchmark for enrollment with 70% (75/107) of eligible participants signing up and an additional 8% (9/107) expressing interest but being placed on a waitlist due to study spaces being filled. Of the 75 students who signed up, 68 attended a meeting to install the ODIN app (68/107, 64% of eligible students). Of the 68 study participants, 64 completed the follow-up survey (94%). To further assess feasibility, average participant compliance (Figure 1) was examined. At all timepoints, average compliance was above the benchmark threshold, with an overall average adherence rate of 90% (median 93%, SD 0.13%, maximum 100%, minimum 0%). Overall, people were asked an average of 339.9 questions (SD 126.92) during the study, and answered an average of 315.9 (SD 122.61) questions. Overall, 65 of the 68 (96%) participants met our benchmark of completing at least 80% of questions asked. One participant received four questions and then did not respond to further questions or attempts to contact them, and they were excluded from subsequent analyses.

**Figure 1:**
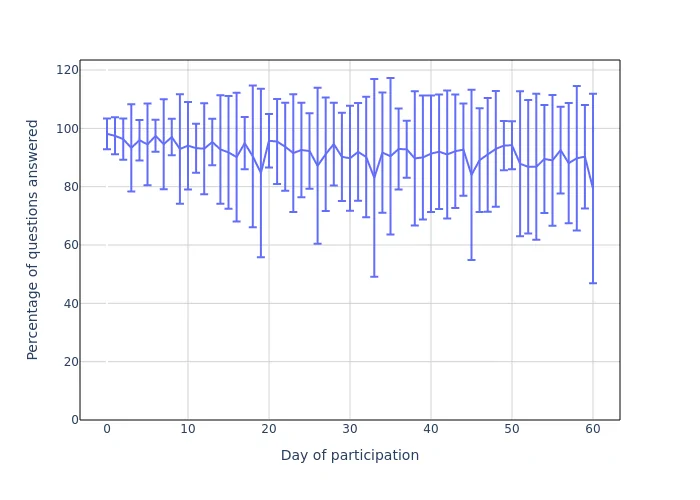
Study compliance overtime. Maximum number of drinks refers to the maximum number of drinks on a single occasion. Survey responses reflect post EMA self reports of drinking during the past 60 days.

#### Compliance Based on Sensor Type (Time vs GPS)

The analysis comparing compliance based on sensor type used to trigger questions revealed that participants’ compliance rates differed as a function of question type, with higher compliance for time-based (mean 87%, SD 16%) compared to location-based questions (mean 76%, SD 27%; t_59_=3.65; *P*<.001).

#### Association Between Compliance and Participants’ Alcohol Consumption

There were high rates of compliance for both morning (85%) and evening (87%) alcohol assessments, and there were 362 person-level drinking days observed. Participants’ average consumption on a given night varied from 1.7 to 11.5 (mean 5.16, SD 2.07) drinks. The logistic mixed model (adjusted intraclass correlation coefficient [ICC]=0.28) explained a moderate amount of variance in compliance (conditional *R*^2^=0.29); however, only a small amount was explained by fixed effects alone (marginal *R*^2^=0.01). The number of drinks a participant reported consuming on a given night (assessed the following morning) was not related to compliance for that night (OR 0.94, 95% CIs 0.80-1.10). Further, participants’ average number of drinks consumed was not associated with their typical compliance (OR 0.92, 95% CIs 0.73-1.17).

If a participant indicated that they had drank alcohol the previous evening, they were asked if at any point they felt too drunk to respond to ODIN questions. Overall, 79.1% of the time (568/718 morning reports) participants indicated that they were never too drunk to respond to EMA questions. When participants indicated that they did become too drunk to respond, this most commonly occurred between 10 PM and midnight (59/718, 8%) and after midnight (56/718, 8%).

#### Compliance and Privacy Concerns

Location privacy concern was not associated with participants’ overall compliance rates, *r*_65_= 0.07, *P*=.57. Both greater general privacy risk awareness (*r*_65_=0.24; *P*=.0497) and privacy concerns (*r*_65_=0.25; *P*=.04) were associated with higher compliance rates.

### Data Quality

#### Prompt Latency

We examined the amount of time between a question prompt and participants answering the question. Looking at all types of questions prompts, on average participants took 7.1 minutes to respond. We then explored response times specifically to questions that were the first in a chain of prompts, for these questions response times were slower (mean 23.8, SD 9.8 minutes).

#### Prompt Delivery

In addition to examining compliance (i.e., participants’ response rate to questions they received), we also examined data quality and missingness in terms of failure to receive anticipated question prompts. We were not able to calculate the exact total number of questions each participant should have received for a number of reasons. This included geolocation questions not being predictable and participants' responses to prompts determining if and how many additional questions they received in a chain. Therefore, we estimated the total number of first questions in time-based chains that each person should have received during the study (20 per week or 172 across the 60 days) and compared this to the number of time-based first in chain questions received. On average, participants received less than the minimum number of prompts (median 143, IQR 110.5-161.0), receiving on average 83% of the time-based question chains. In most cases, it was not possible to determine why the prompts were not received, though it may have reflected phones being turned off or phones losing network connection. In addition, we did experience a technical issue related to daylight savings that impacted prompts being delivered for a number of days after the time change. In total, 19 participants had to relaunch or reinstall the app and this impacted data collection for a period of 1 to 10 days (mean 5.5, SD 3.6 days). One participant received a new phone during the study period and went 2 days without receiving questions before rejoining the study. A second student reported that their phone broke during the study period and that they had not taken the time to have the app set up on their new phone.

#### Concurrent Validity

As a preliminary exploration of the quality of drinking self-report data, we examined the correlation between self-reports of alcohol use post-EMA with EMA drinking data (Table 1). Participants’ retrospective estimates of their average alcohol consumption on a single occasion during the 60-day EMA period were positively correlated with their average drinks per occasion reported in the EMAs (*r*_62_=0.71; *P*<.001). Similarly, participants’ retrospective estimates of how much they consumed on their maximum drinking occasion during the study period were positively associated with the maximum number of drinks observed in the EMA data (*r*_62_=0.71; *P*<.001). These positive associations lend some support to the validity of the EMA alcohol data.

**Table 1:** Correlations between ecological momentary assessment alcohol reports and post-EMA self-report.

	Values, mean (SD)	EMA^a^ average drinks per occasion	EMA maximum number of drinks^b^	Survey average drinks per occasion^c^	Survey maximum number of drinks^c^
EMA average drinks per occasion	5.1 (2.0)	—^d^			
EMA maximum number of drinks	8.4 (3.8)	.83^e^	—^d^		
Survey average drinks per occasion	4.5 (2.2)	.71^e^	.39^f^	—^d^	
Survey maximum number of drinks	9.5 (4.7)	.77^e^	.71^e^	.58^e^	—^d^

^a^EMA: ecological momentary assessment.

^b^Maximum number of drinks refers to the maximum number of drinks on a single occasion.

^c^Survey responses reflect post EMA self-reports of drinking during the past 60 days.

^d^Not applicable.

^e^*P*<.001.

^f^*P*<.01.

### Acceptability

#### Survey Data

Across measures we consistently approached or met benchmarks for acceptability (≥80% endorsement). For example, the vast majority of participants stated that answering questions daily was easy to extremely easy (55/66, 84%) and that using the ODIN app fit somewhat easily to very easily into their routine (51/66, 78%). Overall, 72% (47/66) stated they were moderately to very satisfied with the ODIN app, 88% (58/66) stated they were willing to complete another ODIN EMA study, and 88% (58/66) said that they would recommend being part of an ODIN study to their friends. In terms of emotional responses to using the app, ratings of annoyance (mean 3.84, SD 1.14) did not significantly differ from the neutral midpoint (4; t_63_=–1.10; *P*=.28, Cohen *d*=–0.14). Ratings of interest in the app (mean 4.55, SD 1.26) and enjoyability of using the app (mean 4.58, SD 1.23) were significantly higher than the neutral midpoint (both *P*<.001, Cohen *d*=0.44 and 0.48 respectively), indicating that participants tended to have slightly positive responses to the app.

When asked specifically what difficulties interfered with answering the daily questions, very few participants (6/66, 9%) noted concerns about privacy and data safety. More common difficulties included issues with questions (ie, too many questions or questions at inconvenient times; 27/66, 41%), social reasons (ie, busy schedule, interfering with school work; 33/66, 46%), or technical reasons (eg, app bug related to daylight savings, battery drain; 37/66, 56%).

#### Open-Ended Data

Open-ended responses suggested there were several reasons students found the experience positive. In addition to receiving incentives, participants (21/66, 32%) described that the EMA made them more conscious of their drinking habits. For example, one student stated that, “I was more aware of how many drinks I was consuming on a nightly basis” and another reported, “I thought more about what an appropriate amount of drinks to have was.” Some students also suggested that participating in the EMA changed their drinking behavior in beneficial ways. One student stated that the prompts “would remind me [of] tactics to use before going out and drinking alcohol at night” and another student reported that, “It helped with drinking habits. I was able to control my alcohol better and reflect on how I drank the night before.”

Some students reported that they found answering the questions enjoyable or fun to answer (15%; 10 / 66). These comments often referred specifically to the 10pm evening questions that varied nightly in terms of content. For example, one student stated that “the questions that came sporadically and focused on a variety of topics about student experience… were engaging.” and another said, “Unscrambling the words was random but enjoyable.” At the same time, they appreciated consistency in the timing of questions (11/66, 17%). For example, one student stated, “It was good that the times were predictable” and another said that they were satisfied with their experience because questions were “Routine and easy to predict.”

## Discussion

### Principal Findings

This study adds to the literature by examining the feasibility and acceptability of a 60-day alcohol-related EMA that integrated the use of passive sensors (GPS, Bluetooth, and motion) and triggered questions based on GPS location data. Further, the study examined the role of both individual-level predictors (alcohol use and privacy concerns) and question type in relation to compliance. The study provides support for both the feasibility and acceptability of this approach among college students who drink regularly. The results suggest that college students do not have significant concerns about sharing passive data. Indeed, acceptability ratings suggested that participants generally had a positive experience using the ODIN app and would be willing to take part in a similar study in the future. Further, compliance rates remained high across the 60-day study period, suggesting that it is feasible to conduct this type of EMA for more than a month and maintain engagement.

### Predictors of Compliance

In addition to examining overall feasibility and acceptability, this study examined predictors of compliance rates. Past research has suggested that alcohol use may interfere with students’ responses to EMA questions [23,25]. Our findings are consistent with research suggesting that alcohol use at both a trait [24] and state-level [22,26] are not associated with EMA compliance. Participants’ subjective self-report data suggested that the majority of the time participants did not perceive their alcohol use to interfere with their responses. These findings are somewhat surprising given that alcohol use can impact people’s cognitive capacity in ways that reduce the ability to perform intentional behaviors or engage in deliberative information processing [43]. The lack of impact of alcohol on compliance could be explained by alcohol myopia theory [44], which suggests that once alcohol is consumed people will become more focused on the salient aspects of their immediate environment. Given that the vast majority of the sample reported typically using their cell phones at bars and parties to engage in a number of activities (eg, texting, instant messaging, and taking pictures and/or video), it is possible that cues or prompts to complete EMA surveys are salient and influential for intoxicated people who are already engaging with their phones. The questions participants were asked to complete in this study did not require complex information processing or decision making. Based on the dual-process model [43], it is possible that the impact of alcohol use on compliance may become more pronounced in EMA studies that require participants to complete more novel and effortful tasks.

To our knowledge, this study is the first to examine the relationship between privacy concerns and EMA participation. Given that the study included passive sharing of sensor data, including GPS data, it is notable that location privacy was not related to overall compliance. Furthermore, privacy concerns and awareness were unexpectedly associated with higher compliance rates. Research among community samples of people who use drugs suggests that privacy concerns may be perceived as a barrier to compliance for EMA studies using GPS data [28]. The current findings suggest that the impact of privacy concerns may be less significant for college students reporting on their alcohol use. In this study, participants met with the researchers in order to review consent forms and be trained on the app. While this approach is more labor intensive than participants reviewing consent forms independently, it may be beneficial for ensuring participants feel comfortable responding and that they understand the steps taken to ensure their confidentiality. It is also possible that privacy concerns were not associated with decreased compliance because these concerns are more relevant to the decision to take part in a research study and are not as relevant to responding once in a study [30]. As collecting data on privacy concerns of those who chose not to participate may be challenging, further research using hypothetical EMA designs [45] could help determine if privacy concerns differentially affect willingness to enroll in EMAs that involve passive sensing compared to those that do not.

While participants in this study had higher compliance rates for questions triggered by time rather than location, compliance rates for GPS-triggered questions were still above our benchmark. The difference in response rate by question trigger is consistent with past research that indicates that when questions are more unpredictable, response rates are generally lower [25]. In this study, participants were not specifically told what locations would trigger questions. To improve compliance, it may be beneficial to have participants list out bars or clubs that they attend and create a plan or an implementation intention [46] to check their phone for questions when they are in that specific location. Using participant generated locations could also increase the predictability of where location-based questions are triggered. While consistency may be useful for compliance, the current findings also suggest that it may be beneficial to consider ways to balance asking questions at predictable times with variety in question content to promote participant interest and engagement in EMAs over longer periods. We note that while the less predictable timing of geolocation-triggered questions may have contributed to their lower response rate, the absence of independent geofence validation means that we cannot distinguish this explanation completely from effects related to geofence performance or location misclassification.

### Limitations

This study has a number of limitations. First, it is possible that the sample is not representative of college students who drink frequently due to a self-selection bias of those more comfortable with an EMA involving passive sensors. While this concern is somewhat diminished by the variability in baseline privacy concerns and the high rates of interest among eligible students, it is possible that the sample underrepresented students who are cautious sharing their data. Furthermore, privacy concerns were only measured at baseline and not as part of the EMA protocol. It is possible that participants’ privacy concerns fluctuate or are more salient in some contexts compared to others. While repeatedly asking students about their privacy concerns could itself potentially influence these concerns, longitudinal assessment of context-specific privacy concerns could help better understand EMA responding.

While the current findings suggest that alcohol use did not impact compliance, we did not explore whether the accuracy or quality of participants’ responses decreased as their alcohol consumption increased. The preliminary data supporting the validity of the EMA drinking reports based on comparisons with follow-up survey recall of alcohol use does somewhat address this concern; however, additional research examining how alcohol use impacts the reliability of responses is warranted.

This study focused on human factors that impacted compliance, rather than the impact of technical issues on responding. That said, the generally high compliance and acceptability rates should be interpreted in the context of the study protocol and technical challenges experienced during the study. For example, despite issues related to daylight savings that caused data collection disruptions, participants remained generally satisfied with the EMA experience. It is possible that because most participants did not receive the minimum expected number of time-based prompts, participant fatigue was reduced and this enhanced overall compliance. Finally, past research suggests that financial incentives impact compliance rates [16,31] and therefore the high rates of compliance observed in this study may not extend to research that does not employ similar incentive structures.

We did not conduct an independent ground-truth validation of point-level GPS accuracy or geofence classification. Consequently, some prompts may have been generated while a participant was near, rather than inside, a target venue, and some actual visits may not have generated a prompt. The location-triggered compliance estimate should therefore be interpreted as responsiveness to prompts generated by the geofencing protocol, rather than as confirmation that every response was completed inside a bar or club.

### Conclusions

The integration of passive sensors into college-alcohol EMAs provides an opportunity to study the influence of context on alcohol beliefs and behaviors. The current results indicate that students were generally willing to respond to alcohol-related prompts and questions generated by geofences surrounding bars and clubs. Compliance was not associated with trait-level or in-the-moment alcohol use and was positively associated with some forms of privacy concerns. Response rates were higher for more consistent time-based questions compared to more variable location-based questions. Overall, the current study provides data suggesting that alcohol-related EMAs among college students that incorporate the use of passive sensors can be both acceptable and feasible.

## Data Availability

The datasets generated or analyzed during this study are available from the corresponding author on reasonable request.

## Abbreviations

BLE: Bluetooth Low Energy
CFIP: Concern for Information Privacy
CREMAS: Checklist for Reporting Ecological Momentary Assessment Studies
EMA: ecological momentary assessment
ICC: intraclass correlation coefficient
OR: odds ratio
